# 543. Effectiveness of Black Light in Early Detection of *Pseudomonas Aeruginosa* in Chronic Wounds and Burns

**DOI:** 10.1093/jbcr/irag033.240

**Published:** 2026-04-06

**Authors:** Lourdes Castañon, Elizabeth B McCraw, Joselyn Bobadilla

**Affiliations:** University of Arizona, Tucson, AZ; Banner University Medical Center, Tucson, AZ; Banner University Medical Center, Tucson, AZ

## Abstract

**Introduction:**

*P. aeruginosa* commonly colonizes chronic wounds and burns, complicating detection and treatment. Its fluorescent siderophores can be visualized under UV/black light, offering a potential rapid screening tool. Since nurses routinely perform wound care, bedside screening may enhance early detection.

**Methods:**

In this prospective pilot at Banner Health, nurses performed black light exams during routine wound care for adults with chronic wounds (>4 weeks) or burns. Each exam was paired with a standard culture. Data included fluorescence findings, culture results, time-to-result, and workflow factors.

**Results:**

Four patients were enrolled. All showed positive green/cyan fluorescence with high concordance to culture-confirmed *P. aeruginosa*. Black light provided results in ~1 minute versus hours to days for culture. Nurses reported easy integration into care and strong acceptability, with no adverse effects.

**Conclusions:**

Four patients were enrolled. All showed positive green/cyan fluorescence with high concordance to culture-confirmed *P. aeruginosa*. Black light provided results in ~1 minute versus hours to days for culture. Nurses reported easy integration into care and strong acceptability, with no adverse effects.

**Applicability of Research to Practice:**

Assess the feasibility and preliminary diagnostic accuracy (sensitivity, specificity) of bedside black light screening performed by nursing staff, using gold-standard wound culture as the reference.

Evaluate integration into nursing workflow, ease of use, and acceptability among patients and staff.

**Funding for the study:**

N/A.

